# Autonomic Function following Acute Organophosphorus Poisoning: A Cohort Study

**DOI:** 10.1371/journal.pone.0037987

**Published:** 2012-05-24

**Authors:** Sudheera S. Jayasinghe, Kithsiri D. Pathirana

**Affiliations:** 1 Department of Pharmacology, Faculty of Medicine, University of Ruhuna, Karapitiya, Galle, Sri Lanka; 2 South Asian Clinical Toxicology Research Collaboration, Department of Medicine, Faculty of Medicine, University of Peradeniya, Peradeniya, Sri Lanka; 3 Clinical Neuroscience Center, Department of Medicine, Faculty of Medicine, University of Ruhuna, Karapitiya, Galle, Sri Lanka; University of British Columbia, Canada

## Abstract

Autonomic dysfunction after chronic low level exposure to organophosphorus (OP) pesticides has been consistently reported in the literature, but not following a single acute overdose. In order to study autonomic function after an acute OP overdose, sixty-six overdose patients were compared to 70 matched controls. Assessment of autonomic function was done by heart rate response to standing, deep breathing (HR-DB) and Valsalva manoeuvre; blood pressure (BP) response to standing and sustained hand grip; amplitude and latency of sympathetic skin response (SSR); pupil size and post-void urine volume. The patients were assessed one and six weeks after the exposure. The number of patients who showed abnormal autonomic function compared to standard cut-off values did not show statistically significantly difference from that of controls by Chi-Square test. When compared to the controls at one week the only significant differences consistent with autonomic dysfunction were change of diastolic BP 3 min after standing, HR-DB, SSR-Amplitude, SSR-Latency, post-void urine volume and size of the pupil. At 6 weeks significant recovery of autonomic function was observed and only HR-DB was decreased to a minor degree, −5 beats/min [95%CI 2–8]. This study provides good evidence for the lack of long term autonomic dysfunction following acute exposure to OP pesticides.

## Introduction

Acute pesticide poisoning is a major health problem especially in developing countries. In Sri Lanka, the majority of poisoning cases are self inflicted [1]. Organophosphorus (OP) pesticides compounds were involved in 76% of pesticide poisoning [1], [2]. OP leads to four well defined neurological syndromes, namely acute cholinergic crisis, intermediate syndrome, OP induced delayed polyneuropathy (OPIDN) and chronic OP induced neuropsychiatric disorders (COPIND) [3]. The acute cholinergic crisis reflects the acute inhibition of neuronal acetylcholine esterase (AChE). However the mechanisms behind the other syndromes are less well defined.

Studies have shown abnormalities in motor and sensory nerve conduction studies in farm workers exposed to OP [4], [5]. Effects of chronic mixed pesticide exposure on autonomic nerve function were also demonstrated by Ruijten et al. in 1994 [6]. There have been no studies which have looked into autonomic function following acute single exposure to OP.

The objective of this study was to determine whether acute single exposure to OP leads to autonomic dysfunction.

## Materials and Methods

### Ethics Statement

The study was approved by the Ethical Review Committee, Faculty of Medicine, University of Ruhuna, Sri Lanka. Informed written consent was obtained from the patients and the controls. All clinical investigations were conducted according to the principles expressed in the Declaration of Helsinki.

A cohort study was conducted with age, gender and occupation matched healthy controls. Patients with acute OP poisoning were recruited to the study from a tertiary care hospital and a secondary care hospital in the Southern Province of Sri Lanka from June 2008 to April 2010. At the time of recruitment to the study, these subjects either had features of the cholinergic syndrome or had been given atropine in the peripheral units and then transferred to the collaborating hospitals.

The control group was recruited from persons accompanying the patients to the Teaching Hospital, Galle. Age of the controls was matched to ±3 years of the poisoned individuals. The controls were recruited within one month of recruitment of the respective case.

Participants who had neuropathies, diabetes mellitus and who were on long term medication were excluded from the study.

All neurophysiological investigations were carried out by the first author. She was available in the Clinical Neuroscience Center, Faculty of Medicine, University of Ruhuna where the participants were assessed, throughout the week to carry out the one week assessment and during working days to conduct the six week assessment and to assess the controls.

Plasma cholinesterase (ChE) levels were quantified at four hours and twelve hours after the exposure using the modified Ellman method developed by Worek F et al. (1999) [7].

R-R interval based autonomic function tests (heart rate response to standing, heart rate variation during deep breathing and heart rate response to Valsalva manoeuvre), blood pressure response to standing, blood pressure response to sustained handgrip, sympathetic skin response, size of the pupil and residual urine volume were assessed. In the patients, assessments were carried out at one week (first assessment) and six weeks (second assessment) following the exposure to see the progression of the effects. Controls were assessed only once, since they did not have the exposure and it was assumed that the state of autonomic function remainined the same for the next six weeks.

When performing R-R interval based autonomic function tests, electrodes were attached on the anterior chest wall of the participant. Then continuous electrocardiogram was performed. R-R intervals were analyzed by autonomic nervous system testing software (Neuropack S1, QP948BK).

### Heart rate response to standing

Participants were asked to stand up unaided from lying down position as quickly as possible when performing heart rate response to standing. The heart rate response was expressed by the 30∶15 ratio; ratio of longest R-R interval around the 30^th^ beat after standing to shortest R-R interval around the 15^th^ beat after standing [8]–[10].

### Heart rate response to deep breathing

To perform heart rate variation during deep breathing, participants were asked to breathe deeply and evenly at 6 breaths per minutes (5 seconds in; 5 second out) for three cycles (30 seconds). Maximum and minimum R-R intervals were analyzed during each cycle and converted to beats per minute. Greatest heart rate differences (expiratory-inspiratory difference; E-I difference) during each cycle were calculated and the three differences averaged [10], [11].

### Heart rate response to Valsalva manoeuvre

During the Valsalva manoeuvre, the participant breathed in to a mouth piece connected to a modified sphygmomanometer and maintained an expiratory pressure of 40 mmHg for 15 seconds. The ratio of the longest R-R interval within 20 beats of ending the test to the shortest interval during the test was analyzed. The test was performed three times and the ratios from three Valsalva attempts averaged [10], [11].

### Blood pressure response to standing

Blood pressure was measured while the participant was lying down quietly and around three minutes after standing [11] by using an OMRON SEM-1 automatic blood pressure monitor that uses the oscillometric method of blood pressure measurement. The monitor detects blood movement through the brachial artery and converts the movements into a digital reading.

### Blood pressure response to sustained handgrip

In blood pressure response to sustained hand grip, the participant was asked to press a hand dynamometer with full strength. Then the hand grip was maintained at 30% of the maximum strength for five minutes. Due to muscle contraction, sympathetic activity and vasoconstriction should lead to a rise in blood pressure. The diastolic blood pressure at the end of the effort should be at least 16 mmHg higher than before the manoeuver. An increase in the diastolic blood pressure by 10 mmHg or less is considered as abnormal [10], [11].

### Sympathetic skin response (SSR)

Sympathetic skin response measures the skin potential which is evoked by stimulation (electric, auditory and visual). This potential shows the functioning of the sympathetic nerves of the sweat glands [12]. Participant's skin was cleaned where the electrodes were attached to remove any moisture and gel from the skin. The active electrode was pasted on the center of the palm of the dominant hand. The reference electrode was pasted on the center of the back of the same hand. The ground electrode was attached between the active and the stimulating electrodes. The stimulating electrode was placed on the median nerve of the recording wrist and stimulated with the intensity of 25 mA. Six stimulations were administered with at least 30 sec intervals between each stimulation. The latency was measured from the onset of stimulus artifact to the onset of negative deflection. The amplitude was measured from the baseline to negative or positive peak whichever was the highest. Average latency and maximum amplitude were recorded.

Table 1 shows the cut off values of autonomic function tests [11].

**Table 1:** Cut-off values of autonomic function tests [11].

Test	Normal	Borderline	Abnormal
*The tests reflecting cardiac parasympathetic integrity*			
Heart rate response to Valsalva manoeuvre – Valsalva ratio (VM)	≥1.21	1.11–1.2	≤1.10
Heart rate variation during deep breathing (DB) (breaths per minute)	≥15	11–14	≤10
Heart rate response to standing – standing ratio (ST)	≥1.04	1.01–1.03	≤1.00
*The tests reflecting sympathetic integrity*			
Blood pressure response to standing (mmHg)	≤10	11–29	≥30
Blood pressure response to sustained handgrip (mmHg	≥16	11–15	≤10

### Size of the pupil, pupillary response to light and accommodation

Pupil size was measured using a pupil scale in low light conditions, a card with a series of black circles ranging from 2 mm to 9 mm in diameter. Readings from the pupil scale were obtained by directly comparing the circular scale to the pupil being measured. The pupil scale is routinely available and currently is the primary tool for measuring pupil size in clinics around the country [13]. Reaction to light was assessed by asking the participant to look ahead at a distant object in a low light condition and shining a bright light into each eye (moving from the outer corner of each eye towards the pupil). The accommodation reaction was assessed by asking the patient to look ahead into the distance and then at a finger brought up close to the eye.

### Residual urine volume

Post void residual urine volume was measured ultrasonically after a voluntary void [14], [15].

Data in the first assessment of the patients and the controls were analyzed with unpaired T-test. First vs second assessments were analyzed with paired T-test (GraphPad prism 4). Multiple linear regression model was used to adjust for potential covariates (alcohol consumption, smoking, pralidoxime therapy) and severity of poisoning (the lowest plasma ChE enzyme level, type of OP ingested, Glasgow Coma Scale (GCS) on admission).

## Results

**Figure 1:**
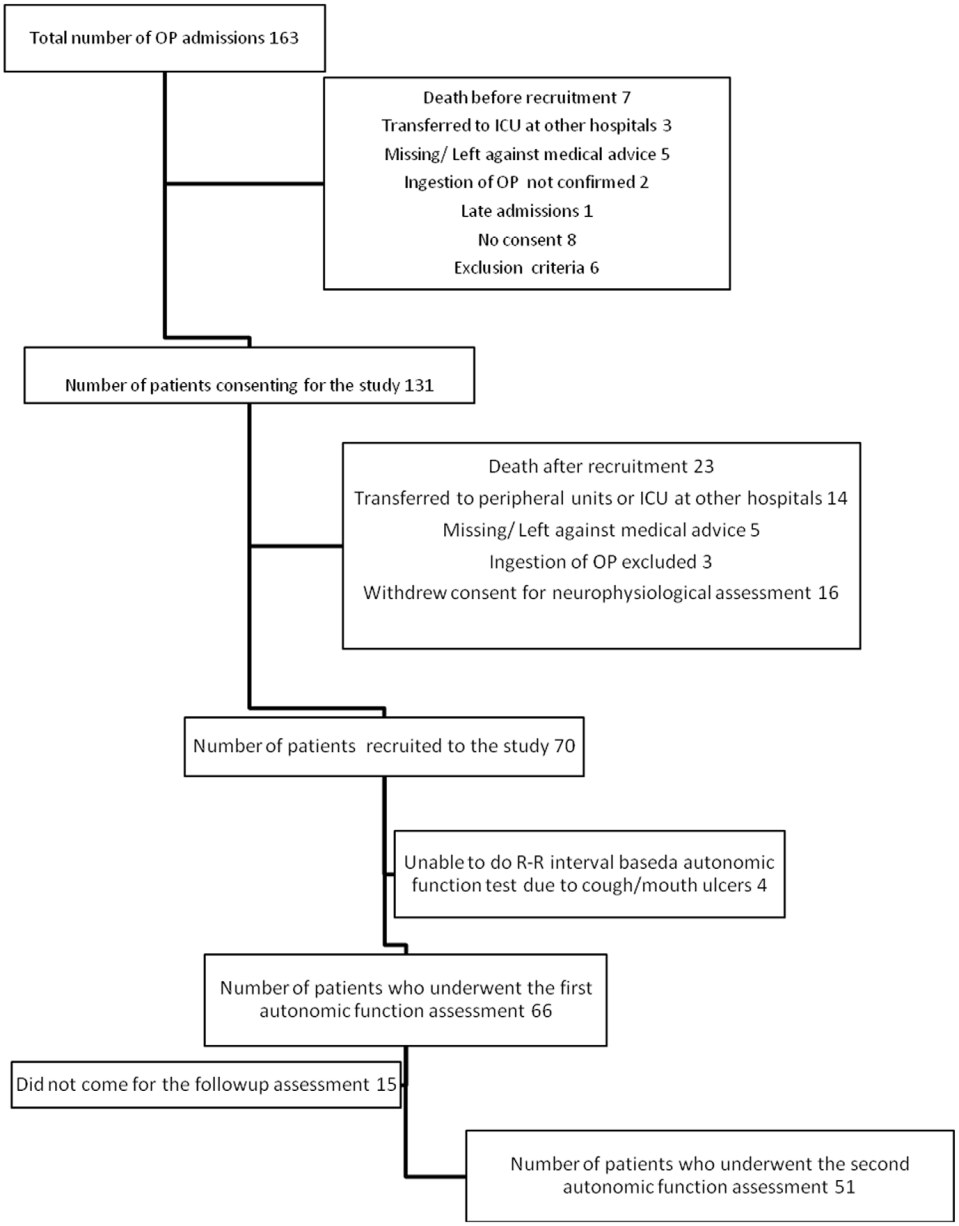
Recruitment of the participants to the study.

Out of a total of 163 OP poisoning admissions to collaborating hospitals, 70 patients were recruited to the study. Four patients were not able to perform autonomic function tests due to cough and mouth ulcers. Sixty-six patients undertook the initial autonomic function assessment. Fifty one patients came for the second assessment at six weeks following the exposure (Figure 1). The control group consisted of 70 participants.

The mean age, gender distribution, category of occupation according to international standard classification of occupation (ISCO-88) by International Labor Organization, smoking habits and pattern of alcohol consumption of the participants are shown in Table 2. None of the patients or the controls had been previously chronically exposed to OP or any other pesticides.

**Table 2:** Descriptive data of the participants.

Descriptive data	Patients	Controls
Age (years)©	32 (12)	33 (12)
Gender (males)	47/66	51/70
Height (m)©	1.59 (0.08)	1.59 (0.11)
Weight (kg)©	55.94 (10.98)	54.69 (9.45)
Body mass index (kg/m^2^)©	22.30 (4.33)	22.10 (6.84)
Occupation		
Student	5	10
Unemployed	8	5
House wife	6	10
Armed forces	2	1
Professionals	5	6
Technicians & associate professionals	0	1
Service workers and shop & market sales workers	2	1
Skilled agricultural and fishery workers	1	0
Craft and related trades workers	3	3
Plant and machine operators and assemblers	7	10
Elementary occupation (eg. selling goods on streets, door keeping and property watching, cleaning, washing, working as laborers in the fields etc.)	25	23
Smoking habits		
Non smokers	32	43
Yes-Regular	13	2
Yes - Occasional	21	25
Alcohol consumption		
None	36	43
Yes-Regular	14	2
Yes-Occasional	16	25

© values are mean (SD).

Table 3 indicates the number of patients according to ingestion of a specific type of OP.

**Table 3:** Number of poisoned cases by type of OP.

Type of OP	Number of poisoned cases
Chlorpyrifos	26
Dimethoate	12
Profenofos	4
Diazinon	3
Malathion	2
Fenthion	1
Acephate	1
Unknown	17

**Table 4:** Autonomic function assessment in patients and controls.

Autonomic function	Patients		Control groupn = 70	Controls vs 1^st^ assessment		Controls vs 2^nd^ assessment		1^st^ assessment vs 2^nd^ assessment	
	First assessmentn = 66	Second assessmentn = 51		Mean change (patients-controls)	95% CI (patients - controls)	Mean change (patients - controls)	95% CI (patients - controls)	Mean change (2^nd^ - 1^st^ assessment)	95% CI (2^nd^ - 1^st^ assessment)
*Blood pressure response to standing*									
Change of SBP 3 min after standing (mmHg)	−4 (−3 to 10)†	−3 (−4 to 7)†	−2 (−3 to 7)†	−2.6	−5.5 to 0.4	1	−4.1 to 2.5	1.3	−4.0 to 1.4
Change of DBP 3 min after standing† (mmHg)	−3 (−8 to 1)†	−6 (−11 to −1)†	−7 (−12 to −4)†	4.6‡	2.4 to 6.7	0.9	−3 to 1.3	−3.2‡	1.1 to 5.3
*Heart rate variation during*									
Standing ratio	1.31 (0.31)	1.27 (0.19)	1.34 (0.28)	−0.04	−0.06 to 0.13	−0.07	−0.02 to 0.2	−0.07	−0.02 to 1.7
Deep breathing (breats/min)	22 (12)	22 (8)	27 (10)	−5‡	−1.6 to −8.8	−5‡	2 to 8	−0.98	−4.1 to 2.2
Valsalva ratio	1.59 (0.34)	1.64 (0.36)	1.69 (0.32)	−0.10	−0.02 to 0.22	−0.07	−0.06 to 0.02	0.05	−0.2 to 0.1
*Sympathetic Skin Response*									
Latency (ms)	1639 (234)	1596 (196)	1543 (152)	96‡	28 to 163	−53	−126 to 2	−56	−39 to 152
Amplitude (mV)	0.51 (0.49)	0.99 (0.69)	1.25 (0.98)	−0.7‡	−1.01 to −0.46	−0.3	−0.005 to 0.6	0.5‡	−0.7 to −0.3
*Residual urine volume* † *(ml)*	22 (0 to 10)†	-	5 (0 to 10)†	17‡	9 to 25	-	-	-	-
*Pupil size (mm)*	4.1 (1.1)	3.1 (0.6)	3.1 (0.3)	1‡	1.30 to 0.72	0.1	−0.3 to 0.09	−1‡	0.6 to 1.4

Values are in mean (SD) except

† median (interquartile range),

‡ significant at 0.05 level (2-tailed).

**Table 5:** Results of the blood pressure response to sustained hand grip.

Blood pressure response to sustained hand grip	patients		Controls	Controls vs 1^st^ assessment		Controls vs 2^nd^ assessment		1^st^ assessment vs 2^nd^ assessment	
	First assessment	Second assessment		Mean change (patients - controls)	95% CI (patients - controls)	Mean change (patients - controls)	95% CI (patients - controls)	Mean change (1^st^ - 2^nd^ assessment)	95% CI (1^st^ - 2^nd^ assessment)
Maximum voluntary pressure (kg)	25.7 (10.3)	25.4 (10.2)	27.7 (9.4)	−2	−1.5 to 5.6	−2	−1.5 to 5.5	0.3	−4.3 to 0.1
Increment of DBP ≥16 mmHg within 5 min	26/50	30/51	55/70	3.38$ ‡	1.4 to 8.1	2.57$ ‡	1.1 to 6.2	1.32$ ‡	0.6 to 3.1

$ odds ratio,

‡ significant at 0.05 level.

**Table 6:** Correlation matrix to identify multicollinearity.

Variables	Glasgow Coma Scale on admission	Lowest AchE activity after four houra of ingestion (µmol/l/min)	Type of OP ingested	Pralidoxime therapy	Smoking habits	Alcohol consumption
Glasgow Coma Scale on admission	-	−0.3 (0.2)	−0.06 (0.6)	−0.1 (0.3)	0.3 (0.006) ‡	−0.05 (0.6)
Lowest AchE activity after four hours of ingestion (µmol/l/min)	-	-	−0.3 (0.1)	−0.1 (0.5)	−0.3 (0.4)	0.2 (0.2)
Type of OP ingested	-	-	-	0.05 (0.6)	−0.2 (0.1)	−0.2 (0.2)
Pralidoxime therapy	-	-	-	-	0.1 (0.3)	0.08 (0.5)
Smoking habits	-	-	-	-	-	0.7 (<0.001) ‡
Alcohol consumption	-	-	-	-	-	-

Analyzed with Spearman's correlation. Values are Spearmans's rho (P value),

‡ significant at 0.05 level.

All patients received atropine and 42 patients received pralidoxime. The patients were atropinised with 0.6–3 mg of atropine initially and then an infusion given to control cholinergic syndrome. The patients received 1–2 g of a loading dose of pralidoxime and maintenance dose of 8–10 mg/kg/hr with a mean of 1.3 (0.7) days. Five patients received only a single dose of maintenance therapy of pralidoxime whereas the maximum duration the patients received the maintenance dose of pralidoxime was 4 days.

Quantification of plasma ChE activity was available in 34 patients either four or twelve hours after the exposure, or both. The median and inter quartile range of plasma ChE activity at four and twelve hours after the ingestion were 886 (155–2607) µmol/l/min and 493 (138–3254) µmol/l/min respectively.

Median inter quartile range of premortem ChE activity in dead patients was 284.5 (31.4–413.9) µmol/l/min. When the value was compared with the lowest ChE activity of patients (median (inter quartile range) 492.6 (138.5–3253.7 µmol/l/min) who underwent autonomic function assessment, there was no significant difference (P = 0.9 by Mann-Whitney U test).

Statistically significant autonomic dysfunction was seen in the first assessment of the patients compared to the controls in change of diastolic blood pressure (DBP) 3 minutes after standing, HR-DB, SSR-amplitude, SSR-latency, post-void urine volme and size of the pupil (Table 4). At six weeks recovery of autonomic dysfunction was observed except in HR-DB (Table 4).

When the number of patients with abnormal autonomic function test was compared with the number of controls with abnormal autonomic function tests (by Chi-Square test) in relation to standard cut-off values of autonomic function tests, no statistically significant difference was observed.

Sixteen patients were not able to perform blood pressure response to sustained hand grip due to painful cannula site on their dominant hand. Twenty-four patients were not able to complete the test. Twenty-one patients in the second assessment and 15 controls were also not able to complete the test. Participants (26 in the first assessment, 30 in the second assessment and 55 in the controls) who could complete the test were within the normal limits (Table 5). However odds ratio of the number of patients who were able to complete the test in the first assessment (vs controls) and the second assessment (vs controls) were 3.4 and 2.3 respectively.

The complex of SSR was almost flat in seven patients in the first assessment and in one patient in the second assessment. None of the controls showed flat SSR complex.

There were no abnormalities seen in the pupillary response to light and accommodation.

The urinary bladder of most patients was catheterized. The catheter was removed only at the time of discharge from the hospital. Hence residual urine volume was measured in 27 patients on discharge and 24 matched controls (Table 4).

In the correlation matrix, it was observed that none of the bivariate correlations are highly correlated except alcohol consumption and smoking habits, GCS on admission and smoking habits (Table 6). To determine whether any multicollinearity were present, and to understand whether there is a strong linear association between each predictor variable and all other remaining predictors, the Variance Inflation Factor (VIF) was examined. None of the VIF exceeds 10.

Adjusted multiple linear regression revealed significant effects of alcohol consumption on change of SBP 3 min after standing (B = 4.4, SE = 1.6, p = 0.009) and size of the pupil (B = −0.6, SE = 0.3, p = 0.04); and the effects of smoking on residual urine volume (B = 11.8, SE = 5.1, p = 0.03) and size of the pupil (B = 0.5, SE = 0.2, p = 0.04).

## Discussion

The comparison of the patient's response vs controls, to the cut-off values of autonomic function tests showed no abnormal findings. The current study did not reveal persistent autonomic dysfunction following acute single ingestion of OP compared to the matched controls.

Although autonomic function in farm workers has been looked in to, there were no studies that looked at autonomic function following acute OP exposure. In the current study neurophysiological parameters of the exposed individuals were compared with the parameters of matched controls.

Variation of heart rate during rest, deep breathing and isometric muscle contraction were tested in flower bulb farmers who had chronic mixed pesticide exposure by Ruijten M.W.M.M et al. (1994). Significant autonomic dysfunction was shown in variation of heart rate during rest and deep breathing [6]. We did not assess variation of heart rate during rest but results for heart rate variation during deep breathing were similar in both studies.

In the tests reflecting parasympathetic damage (heart rate response to Valsalva manoeuver, heart rate variation during deep breathing and immediate heart rate response to standing) a positive result was shown only with heart rate response to deep breathing. It is well known that the response of the heart rate can be abolished by atropine [9]. The first assessment of the patients was conducted within the mean of three (IQR 1–4) days after the cessation of atropine therapy. The effects of atropine may not have disappeared completely even 6 weeks after exposure. On the other hand, only 48/66 patients were able to do the Valsalva manoeuver. The sample size may not have been adequate to draw a conclusion. Or else the autonomic function of exposed individuals may not have been affected.

Change of diastolic BP 3 min after standing was significantly low in the patients, but recovered completely at six weeks after exposure (Table 4).Blood pressure response to standing reflects sympathetic function. But it begins to show abnormal results with more severe sympathetic nerve damage [9]. Sympathetic damage in our patients may not have been severe enough to give abnormal results in the blood pressure response to standing.

Reduction of amplitude and prolongation of latency in SSR are in favor of sympathetic damage at one week after exposure. SSR represents the change in voltage measured at the skin surface following a single electrical stimulus. It depends on the electrical activity arising from sweat glands [12]. Latency of SSR is inversely proportional to the number of sweat glands innervated [12]. Hence sympathetic innervations to sweat glands were less in the patients compared to the controls. In the second assessment both the latency and the amplitude of SSR were improved. We could not eliminate the effects of atropine on sweat glands and changes in the first assessment may have been due to effects of atropine. However patients did not show sympathetic dysfunction as reflected in SSR at six weeks after exposure.

It is well known that high residual urine volume is associated with increased risk of urinary tract infections. Since our results showed that residual urine volumes in patients were significantly higher than in the controls, it is very important to follow up such patients further.

There was no change in size of the pupil in the second assessment in the patients compared to the controls. Large pupil size in the first assessment of the patients was probably due to the effects of atropine.

Plasma half life of pralidoxime is about 90 minutes. Therefore pralidoxime is unlikely to remain in plasma even at one week assessment since the maximum duration of receiving pralidoxime by the patients was four days.

One third of patients was admitted with co-ingestion of alcohol. Acute alcohol ingestion is unlikely to have had any effects on the parameters we assessed, since the assessments were carried out at one week after the exposure. It is well known that long term alcohol consumption is associated with neuropathies including autonomic neuropathy. Adjusted multiple linear regression did not show widespread effects of alcohol consumption on autonomic function.

The number of patients who could complete the test of blood pressure response to sustained hand grip was significantly lower than the number of controls who could complete the test. The difference may be due to muscle weakness following poisoning.

Dimethoate active metabolite (omethoate) inhibits acetylcholinesterase (AChE) slowly and butyrylcholinesterase (BChE) activity can be near normal in symptomatic patients [16]. Overall ChE activity in dimethoate poisoned patients may be high. In contrast the active metabolite of chlorpyrifos (chlorpyrifos-oxon) is more potent and inhibits BChE more than AChE [16]. In chlorpyrifos poisoning, all patients with sufficient AChE inhibition to provide clinical symptoms will have markedly decreased BChE activity [16]. Among 26 patients identified as chlorpyrifos ingestion, six patients showed high levels of ChE. All six patients had cholinergic features on admission and were treated with atropine and pralidoxime. High levels of ChE activity in these patients may be due to incorrect identification of the poison or to mixed ingestion. Overall high levels of ChE in our patients could be due to dimethoate poisoned patients.

OP is a well known neurotoxic substance, and OP poisoning produces a variety of neurological syndromes due to various factors [4], [17], [18]. However the current study did not show evidence of persistent autonomic dysfunction following a single acute exposure to OP.

### Limitations

Neurophysiological assessments in our study were done twice, at one and six weeks following the exposure. Although there were 66 patients, all the patients were not able to perform all the tests. Therefore small sample size was a limitation in some assessments leading to curtailed conclusions. We did not evaluate the status and nerve innervations of sweat glands by using a subcutaneous acetylcholine or pilocarpine injection test nevertheless SSR latency and amplitude did not show persistent abnormalities.
